# Gut Microbiota Modulation in the Management of Chronic Obstructive Pulmonary Disease: A Literature Review

**DOI:** 10.7759/cureus.66875

**Published:** 2024-08-14

**Authors:** Srihita Patibandla, Nilay Bhatt, Sean Lief, Samer M Beauti, Ali Z Ansari

**Affiliations:** 1 Department of Internal Medicine, Trinity Health Grand Rapids, Grand Rapids, USA; 2 Department of Internal Medicine, Hospital Corporation of America (HCA) Houston Healthcare Clear Lake, Webster, USA; 3 Department of Internal Medicine, William Carey University College of Osteopathic Medicine, Hattiesburg, USA; 4 Department of Pathology, William Carey University College of Osteopathic Medicine, Hattiesburg, USA

**Keywords:** antibiotics, prebiotics, synbiotics, fiber, probiotics, gut dysbiosis, copd, fecal microbiota transplantation, gut microbiome, chronic obstructive pulmonary disease

## Abstract

Chronic obstructive pulmonary disease (COPD) represents a significant global health burden, characterized by progressive airflow limitation and exacerbations that significantly impact patient morbidity and mortality. Recent research has investigated the interplay between the gut and the lungs, known as the gut-lung axis, highlighting the role of the gut microbiome in COPD pathogenesis. Dysbiosis, characterized by microbial imbalance, has implications for COPD, influencing disease progression and susceptibility to exacerbations. This comprehensive review integrates current scientific literature on gut microbiota modulation as a therapeutic avenue for COPD management. Through a thorough discussion of studies investigating probiotics, prebiotics, synbiotics, antibiotics, dietary fiber, and fecal microbiota transplantation, this review summarizes the influence of these interventions on COPD via the gut-lung axis through the modulation of systemic inflammation, mucosal immunity, and metabolic processes. The interventions highlighted here show potential in preventing COPD exacerbations, preserving lung function, and improving patient quality of life. By compiling the latest scientific evidence, this review provides a comprehensive framework for physicians and researchers to deduce the effectiveness of gut microbiome modulation as an adjunctive therapeutic strategy in COPD management.

## Introduction and background

Chronic obstructive pulmonary disease (COPD) is a progressive inflammatory disorder characterized by pathologic changes in the small and large airways of the lungs [1]. It is a leading cause of death worldwide, with prevalence increasing up to two to three times with age [1]. Furthermore, COPD is predicted to be the cause of 5.4 million deaths annually by 2060, becoming the primary cause of death among various other chronic diseases [2]. COPD exacerbations, characterized by episodes of acutely worsening symptoms, increased airway and systemic inflammation, and physiological changes, are the target of many pharmacological interventions [3]. Triggers of acute exacerbations include respiratory viruses and bacteria that infect the airways and cause an inflammatory response [3]. COPD exacerbations significantly impact patients' quality of life, health status, and disease progression by accelerating the decline of lung function, resulting in frequent hospitalizations, changes in medications, and frequent medical visits, and are ultimately the primary cause of death in COPD patients [2].

Recent research has increasingly focused on the gut-lung axis and the role of the gut microbiome in COPD pathogenesis. The gut microbiome, comprising of trillions of microorganisms inhabiting the gastrointestinal tract, plays a significant role in immune regulation, metabolism, and host homeostasis [2]. The most prevalent organisms in healthy gut microbiomes are Firmicutes, Bacteroidetes, Fusobacteria, Actinobacteria, Proteobacteria, and Verrucomicrobia, and the most common subclasses from these phyla are *Bifidobacterium*, Lachnospiraceae, *Streptococcus*, *Enterococcus*, and *Lactobacillus* [2]. Recent literature reveals gut dysbiosis, disruption of the microbiome, resulting in an imbalance of the normal microbes, to be associated with COPD [4]. Individuals with COPD tend to have a shift in the gut microbiome, which overall seemed to be associated with reduced microbial diversity, an increase in Firmicutes, *Prevotella*, and *Streptococcus*, and a decrease in Bacteroidetes [5]. Gut dysbiosis of the intestinal microbiome has been shown to trigger lung microbiome dysbiosis through changes in circulating inflammatory cytokines by transfer of gut microbes to the airways [5].

Several studies have outlined the mechanisms by which gut dysbiosis influences COPD, particularly through immune modulation and systemic inflammation [6,7]. Gut dysbiosis can lead to an altered gut microbiome, resulting in increased intestinal permeability and translocation of microbial products like lipopolysaccharides (LPS) into the bloodstream. This systemic exposure triggers an inflammatory response, contributing to chronic inflammation seen in COPD. Additionally, the imbalance in gut microbiota can modulate immune responses by affecting T-cell differentiation and cytokine production, further exacerbating lung inflammation and impairing pulmonary function in COPD patients. However, comprehensive literature reviews highlighting the latest interventions that have been explored to target the gut microbiome for the treatment of COPD are lacking per our knowledge. With this background, this review aims to explore the current understanding of gut microbiota modulation as a therapeutic strategy for preventing and managing COPD exacerbations. A comprehensive literature search was performed, focusing on studies investigating probiotics, prebiotics, synbiotics, antibiotics, dietary fiber, and fecal microbiota transplantation (FMT) as adjunctive therapies for improving COPD outcomes. This review highlights the need for developing non-pharmacological interventions as adjunctive mainstream treatments for COPD.

## Review

Methodology

**Figure 1:**
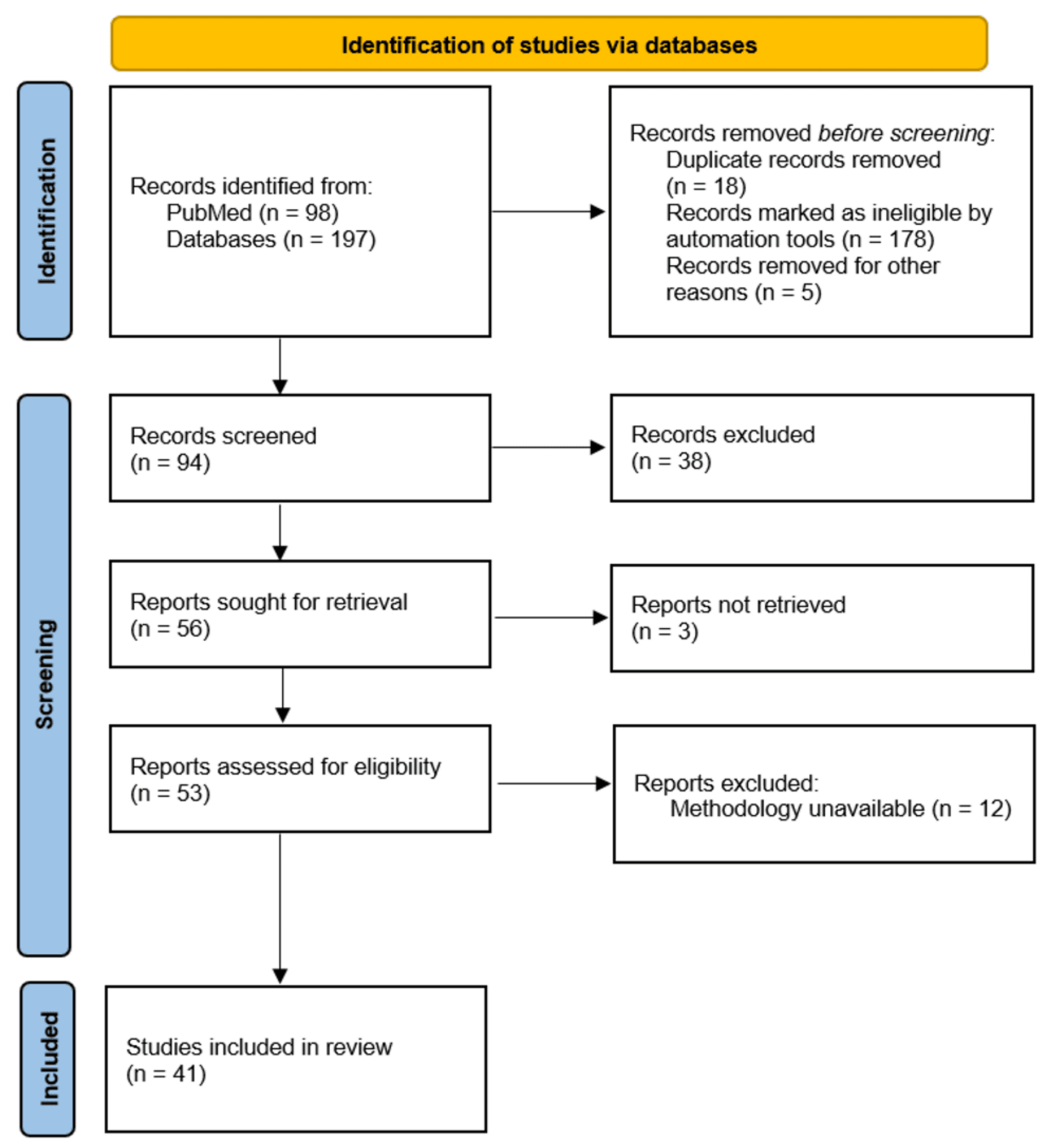
PRISMA flowchart illustrating the process of article selection PRISMA - Preferred Reporting Items for Systematic Reviews and Meta-Analysis

We searched the databases PubMed and Google Scholar by using the following keywords in different combinations: "chronic obstructive pulmonary disease", "COPD", "gut dysbiosis", "gut microbiome", "prebiotics", "probiotics", "synbiotics", "antibiotics", "fiber", and "fecal microbiota transplantation" (last search conducted in July 2024). In the present literature review, we focused our attention on papers discussing the effects of gut microbiota modulation on the prevention and management of COPD exacerbations. We included experimental studies and clinical trials studying the effects of probiotics, prebiotics, synbiotics, fiber, antibiotics, and fecal microbiota transplantation. The inclusion criteria included original articles, systematic reviews, and meta-analyses that appeared to be the most interesting and relevant to the topic of our paper. Studies that were published in languages other than English and studies that were not open access where insufficient information was accessible were excluded. We performed a systematic assessment of the risk of bias for each included study using the Newcastle-Ottawa Scale (NOS). In cases where multiple studies covered the same topic and had similar findings, only the most recently published study was selected for discussion. The selection process of the studies is shown in Figure 1 in the Preferred Reporting Items for Systematic Reviews and Meta-Analysis (PRISMA) flowchart.

Probiotics, prebiotics, synbiotics

Probiotics, defined as live microorganisms that confer health benefits when administered in adequate doses, are widely used to shape the gut microbiome by introducing beneficial bacteria into the gut ecosystem [8]. In contrast, prebiotics are nondigestible food ingredients that selectively promote the growth and activity of beneficial microorganisms, serving as fuel for probiotics and indigenous gut microbiota. Synbiotics are a synergistic combination of probiotics and prebiotic substrates that are selectively used by host microorganisms for growth and thereby modulate the gut microbiome [8]. Usage of these compounds for treatment of gut dysbiosis and COPD management has been studied extensively in the literature.

In a mouse model experiment by Budden et al. [9], probiotic administration of both wildtype and mutant (impaired acetate production capacity) *Bifidobacterium longum* subsp. *longum*, was shown to alleviate cigarette smoke-induced inflammation and prevent depletion of cecal butyrate levels produced by other commensal microbes. In another mouse model of cigarette smoke extract and porcine pancreatic elastase-induced COPD studied by Kim et al. [10], the probiotic *Lactiplantibacillus plantarum* KF511 was shown to reduce pulmonary inflammation, attenuate lung tissue damage and mucin hypersecretion, suppress immune cell infiltration, and decrease the production of matrix metalloproteinases, cytokines, and chemokines via inhibition of the phosphorylation of p38, extracellular signal-regulated kinase (ERK), and c-Jun N-terminal kinase (JNK). The probiotic also inhibited the activation of mitogen-activated protein kinases in the lungs of COPD mice and NCI-H292 human pulmonary mucoepidermoid cells [10]. These studies provide foundational evidence for the efficacy of *Bifidobacterium longum* subsp. *longum* and *Lactiplantibacillus plantarum* KF511 in the management of COPD. However, given the small sample sizes used, further experimental research is needed before research using human participants can be performed.

*Lactobacillus rhamnosus* (*Lr*), another one of the most widely used probiotic strains, has been extensively used in experimental studies. Vasconcelos et al. [11] investigated the effects of *Lr* on lung inflammation and gut dysbiosis in a murine asthma-COPD overlap syndrome (ACOS) model. *Lr* reduced leukocyte population, reduced bronchial hyperactivity, reduced pro-inflammatory cytokines, reduced airway remodeling, decreased Firmicutes species, increased Deferribacteres, decreased *Staphylococcus*, increased *Mucispirillum*, and improved fecal bacterial diversity in ACOS mice [11]. A similar study of *Lr* effects on cigarette smoke-induced COPD mice by Carvalho et al. [12] supported these findings, indicating that the probiotic attenuates the inflammatory response in the airways and lung parenchyma. The authors here also observed in vitro assays of murine bronchial epithelial cells and human bronchial epithelial cells exposed to cigarette smoke and found that *Lr* modulated the balance between pro-inflammatory and anti-inflammatory markers, showing promise in controlling airway inflammation and lung remodeling in COPD [12]. In another experiment by Lal et al. [6], human bronchial epithelial cells were exposed to cigarette smoke extract to simulate COPD effects. Treatment with a probiotic blend of *Lactobacillus* strains AB11, AB12 and AB13 versus control was studied and found decreased levels of matrix metalloproteinase (MMP)-9. In part two of the same study, COPD mouse models treated with the probiotic blend showed reduced MMP-9 mRNA and protein, cytokines, and lung neutrophil counts compared to controls [13]. These studies lay the groundwork for the use of lactobacilli probiotics in COPD treatment.

The therapeutic effects of *Lr* have also been evaluated in a randomized controlled trial by Hua et al. [14], where patients with moderate-to-severe COPD were treated with conventional COPD treatment based on the Global Initiative for Chronic Obstructive Pulmonary Disease (GOLD) 2019 report recommendations, plus either inhaled amikacin, *Lr*, or influenza-*S. pneumoniae* vaccination. Both the influenza-*S. pneumoniae* vaccine and long-term probiotic use were shown to significantly delay the next moderate-to-severe acute exacerbation of COPD in these patients [14]. These studies show promise for the routine use of *Lr* probiotics for prophylactic management of COPD exacerbations.

Other experimental studies investigated the combined effects of *Lr* when supplemented, along with *Bifidobacterium breve* (*Bb*). *Lr* and *Bb* are often used together in probiotics due to their complementary mechanisms of action and synergistic anti-inflammatory effects [15]. In de Sá Fialho et al.'s [16] study, COPD mice were treated with both *Lr* and *Bb*, and together attenuated the cellularity in bronchoalveolar lavage fluid, reduced pro-inflammatory cytokines, reduced anti-inflammatory cytokines, reversed airway remodeling, and reduced the expression of MMP-9, MMP-12, NF-κB, signal transducer and activator of transcription 3 (STAT3), and toll-like receptors (TLRs) 2, 4 and 9 in the lungs. In another study of human bronchial cell cultures stimulated with cigarette extract by Aimbire et al. [17], *Bb* and *Lr* effectively reduced the levels of IL-6, IL-1β, TNF-α, CXCL1, CXCL5, CXCL8, and CXCL9, while increasing IL-10 and TGF-β levels, highlighting their ability to modulate inflammatory mediators.

These findings were followed by clinical studies where lactobacilli and bifidobacteria were combined with steroids and bronchodilators to investigate the effects of combination therapy. Chen et al. [18] published a retrospective analysis where the probiotics were combined with budesonide and ipratropium bromide for the treatment of patients with COPD, and lung function, inflammatory marker levels, airway remodeling, and gut microbiota were studied. The treatment group given budesonide and ipratropium bromide in combination with probiotics had a greater decrease in inflammatory markers, greater improvement in lung function tests, higher levels of lactobacilli and bifidobacteria, and lower levels of Enterobacteriaceae and *Enterococcus* compared the treatment group given budesonide and ipratropium bromide alone [18]. A similar study was performed by Liu et al. [19]; however, the control group was treated with just budesonide alone, without ipratropium bromide. The experimental group received treatment with budesonide and the probiotic containing *Lactobacillus* and bifidobacteria, and in addition to improved lung function and gut microbiota levels as seen in the previous study, patients also showed faster symptom relief and improved quality of life with probiotic treatment [19]. These studies advocate for the use of the combined *Lactobacillus* and bifidobacteria probiotic in the management of COPD and the improvement of patient quality of life. However, studies exploring the medicinal effects of bifidobacteria probiotics alone in patients with COPD are lacking in the literature, and this would be an appropriate area of future research.

Through review of the literature, we also found several animal studies that evaluated the effectiveness of multispecies probiotics. Hu et al. [20] treated COPD rats exposed to cigarette smoke and intratracheal administration of LPS with a traditional Chinese herbal compound called Peitu Shengjin Recipe (PSR) and Biostime Probiotic Powder (Biostime, Hong Kong, China) containing *Lactobacillus helveticus*, *Bifidobacterium infantis*, and *Bifidobacterium bifidum*. Biostime Probiotic Powder and PSR were shown to have protective effects on COPD rats as evidenced by lower resistance of inspiration (RI), reduced inflammatory cell infiltration, intact alveoli, and reversed levels of TNF-α, INF-γ, IL-1β, IL-4, and IL-10 [20]. In another rat COPD model study by Ya Li et al. [21], probiotics containing *Bifidobacterium acidophilus*, *Lactobacillus salivarius*, *Lactobacillus casei*, *Streptococcus thermophilus*, and *Bifidobacterium bifidum* improved respiratory function, ameliorated histopathological damage, decreased inflammation, and improved intestinal mucosal response via the short-chain fatty acids (SCFAs)/GPR43/NLRP signaling pathway [21]. These studies provide preliminary evidence for the usage of various combination probiotics in the routine management of COPD, but further investigation is needed due to low sample sizes.

Clinical studies involving multispecies probiotics include a randomized controlled trial by Karim et al. [22], where the effects of the probiotic Vivomixx 112 billion on sarcopenia and physical capacity in COPD patients were studied. Vivomixx is a probiotic containing *Streptococcus thermophilus*, bifidobacteria, and lactobacilli [23]. The study concluded that the probiotic combination improves muscle strength and functional performance in COPD patients by reducing intestinal permeability and stabilizing the neuromuscular junction [22]. The study provides further evidence for the translatability of findings from animal studies to clinical trials, advocating for the usage of probiotics in clinical practice. Alternatively, Koning et al. [24] published an interesting paper studying how multispecies probiotics impact immune biomarkers during and after antibiotic therapy in COPD patients with acute exacerbations. The probiotic consisted of bifidobacteria, lactobacilli, and *Enterococcus faecium*. However, treatment did not cause any noteworthy changes in biomarkers, and any observed effects were believed to be attributed to the resolution of exacerbation and not due to associated with changes in gut microbiota [24]. While the lack of significance may be attributed to low sample sizes, comparing these studies shows that probiotics seem to have limited use in acute exacerbations and provide more benefits when used long-term as a preventative measure. This idea finds further support in a cross-section study by He et al. [25], where the authors found that a high dietary fiber intake was associated with a lower prevalence of COPD, particularly in smokers, participants aged 40-60 years old, and non-obese participants. Longitudinal randomized controlled studies are needed to confirm whether long-term probiotic supplementation would benefit patients with COPD.

Some papers have also discussed the therapeutic potential of lesser-known microbes such as *Parabacteroides goldsteinii* (*Pg*), *Pediococcus pentosaceus*, and *Bacillus clausii*. Lai et al. [26] isolated *P. goldsteinii* from a COPD murine model, which was shown to improve COPD by reducing intestinal inflammation, enhancing cellular mitochondrial and ribosomal activity in the colon, restoring amino acid metabolism, inhibiting lung inflammation, and antagonizing the toll-like receptor four signalling pathway. Liu et al. [27] demonstrated the efficacy of *Pediococcus pentosaceus* SMM914 (SMM914) in delaying COPD progression and reducing pulmonary oxidative stress by creating a shift in the gut microbiome towards microbes that produce SCFAs and metabolize antioxidants. Younus et al. [28] performed a randomized controlled trial that concluded that the administration of *B. clausii* significantly improved the rate of exacerbations and COPD assessment test (CAT) scores in patients compared to control. No significant difference was seen in C-reactive protein (CRP) levels after treatment. These papers open the doors for further investigation of these microbes as therapeutic options for the prevention of COPD exacerbations. The studies discussed in this section have been summarized inTable 1.

**Table 1:** Selected studies investigating the role of probiotics, prebiotics, and synbiotics in the management of COPD COPD - chronic obstructive pulmonary disease; ACOS - asthma-COPD overlap syndrome

Reference	Subjects	Treatment	Study design	Sample size (N)	Main results
Budden et al. [9]	Female C57BL/6 mice exposed to cigarette smoke or normal air for 8 weeks	Vehicle (PBS + 0.05% L-cysteine) or *B. longum* subsp. *longum* by gavage three times per week	Experimental study	5-6	Both strains of *B. longum* subsp. *longum* helped to reduce lung inflammation, lower the expression of inflammatory cytokines and adhesion factors, and counteracted the depletion of butyrate in the caecum caused by cigarette smoke. Therefore, administering *B. longum* subsp. *longum* as a probiotic, regardless of its acetate-producing ability, effectively alleviated cigarette smoke-induced inflammation and restored cecal butyrate levels.
Kim et al. [10]	NCI-H292 human pulmonary mucoepidermoid cells; Male BALB/c (5 weeks) mice	10 mg/kg of roflumilast (ROF) as the positive control group, 1 × 10^8^ CFU/mouse LPKF511-supplemented (511–8) group, and 1 × 10^9^ CFU/mouse LPKF511-supplemented (511–9) group	Experimental study	5	*L. plantarum* KF511 reduced immune cell infiltration and the production of mucin, matrix metalloproteinases, cytokines, and chemokines in bronchoalveolar lavage fluid. It also mitigated tissue damage and excessive mucin secretion in the lungs. Additionally, *L. plantarum* KF511 inhibited the activation of mitogen-activated protein kinases in the lungs of COPD mice and in NCI-H292 cells.
Vasconcelos et al. [11]	Mice exposed to house dust mite (HDM) and cigarette smoke to induce ACOS	*Lactobacillus rhamnosus* administration to treatment group	Experimental study	-	In *Lr*-treated ACOS mice, leukocyte population, bronchial hyperreactivity, pro-inflammatory cytokines, and airway remodeling were reduced. Similarly, levels of IL-4, IL-5, IL-13, STAT6, and GATA3, as well as IL-17, IL-21, IL-22, STAT3, and RORɣt, decreased after *Lr* treatment. Additionally, IL-2, IL-12, IFN-γ, STAT1, and T-bet, along with IL-10, TGF-β, STAT5, and Foxp3, were restored following *Lr* administration. The treatment also resulted in a reduction of *Firmicutes* and an increase in *Deferribacteres*. Furthermore, *Lr* decreased *Staphylococcus* levels while increasing *Mucispirillum* in ACOS mice, and improved fecal bacterial β-diversity.
Carvalho et al. [12]	Three-month-old male C57Bl/6 mice; Human bronchial epithelium (BEAS) cells	Gavage with probiotic *Lactobacillus rhamnosus* (*Lr*) (1×10^9^ CFU/0.2 mL PBS/mouse) (Liane Laboratory, Ribeirão Preto, SP) each day for seven days prior to COPD induction. Murine bronchial epithelial cells or BEAS cells were incubated with a density of 5×10^5^ /mL of *Lr*	Experimental study	7	*Lr* diminished the inflammatory response in both the airways and lung parenchyma, reducing the infiltration of inflammatory cells and the production of pro-inflammatory markers. It also lowered levels of metalloproteases in lung tissue and decreased lung remodeling. Along with reducing the expression of TLR2, TLR4, TLR9, STAT3, and NF-κB in lung tissue, *Lr* increased the levels of IL-10, SOCS3, and TIMP1/2. In murine bronchial epithelial cells and BEAS cells exposed to cigarette smoke extract (CSE), *Lr* inhibited the production of pro-inflammatory markers. Additionally, *Lr* downregulated the expression of NF-κB and STAT3, while upregulating SOCS3.
Lal et al. [13]	Human bronchial epithelial cells (NHBE); 6-8-week-old C57BL/6 mice	A probiotic blend of live *Lactobacillus* strains AB11, AB12, and AB13 (AB Blend #1, obtained from Alveolus Bio, Inc) versus control	Experimental study	-	In vitro: Cell supernatants from *E. coli*-treated NHBE cells exposed to AB Blend #1 exhibited lower levels of MMP-9 compared to controls. In vivo: In both mouse disease models, treatment with AB Blend #1 led to reduced MMP-9 mRNA and protein levels, as well as decreased neutrophil counts in the lungs, compared to controls.
Hua et al. [14]	Moderate-to-severe COPD subjects per GOLD diagnostic criteria with a history of moderate-to-severe exacerbations in the previous year	Inhaled amikacin (0.4 g twice daily, 5-7 days monthly for 3 months), oral *Lactobacillus rhamnosus* GG (1 tablet daily for 3 months), or influenza-*S. pneumoniae* vaccination	Prospective randomized controlled trial	24-34	Individuals who received dual vaccination and oral probiotics experienced a significantly delayed onset of the next moderate-to-severe AECOPD compared to those who received only conventional treatment. In subjects with a high symptom burden, exacerbations were significantly delayed in the group treated with inhaled amikacin compared to the conventional treatment group. All three interventions appeared to be safe and well-tolerated in patients with stable COPD.
de Sá Fialho et al. [16]	C57Bl/6 mice with COPD induced by cigarette smoke inhalation of 14 cigarette per day, twice a day, 7 times/week for 60 days	The mice were treated with *Lr* and *Bb* at the same time	Experimental study	-	*Bb* and *Lr* reduced cellularity in BALF and decreased pro-inflammatory cytokines while increasing anti-inflammatory ones. The probiotics also lowered the expression of MMP9, MMP12, NF-κB, STAT3, and TLR 2, 4, and 9 in the lungs. Additionally, they altered airway remodeling, affecting inflammatory infiltrate, alveolar enlargement, collagen, and elastic fibers.
Aimbire et al. [17]	Human Bronchial Epithelial (BEAS) Cells Culture Stimulated with Cigarette Extract	*Lr* or *Bb* was added to the BEAS culture	Experimental study	-	Both *Lr* and *Bb* effectively reduced levels of IL-6, IL-1β, TNF-α, CXCL1, CXCL5, CXCL8, and CXCL9, while increasing levels of IL-10 and TGF-β. These probiotics modulate the synthesis of pro-inflammatory mediators, lowering the concentration of cytokines and chemokines secreted in the BEAS cell culture supernatant.
Chen et al. [18]	Patients with COPD aged between 18 and 80 years old	2 mL Budesonide + 1 mL Ipratropium bromide + 2 mL normal saline twice a day; probiotics were administered orally with *Bifidobacterium* *Lactobacillus* triple live bacteria 3 times a day, 2 g/time	Retrospective analysis	59	FVC, MMEF, PEF, and FEV1 showed greater improvement in the observation group compared to the control group. After treatment, serum levels of TNF-α, IL-6, and PCT were reduced in both groups, with the observation group showing lower levels than the control. Serum levels of MMP-9, VEGF, basic fibroblast growth factor, and NGF were also lower in the observation group compared to the control group. Additionally, levels of Lactobacilli and Bifidobacteria increased in both groups with treatment, with the observation group having higher levels, while the levels of Enterobacteriaceae and *Enterococcus* were lower in the observation group.
Liu et al. [19]	COPD patients aged 51-82 years old	Budesonide 1 mg + physiological saline 2.5 ml, administered as nebulized inhalation for 15 minutes twice a day; *Bifidobacterium* *Lactobacillus* Triple Viable Tablets 0.5 g/tablet taken orally 2g/time, three times daily for two weeks	Retrospective analysis	59 control, 65 observation group	After two weeks of treatment, the observation group showed greater improvement in lung function compared to the control group. Compared to budesonide treatment alone, the combination of budesonide with bifidobacteria and lactobacilli resulted in a shorter time to symptom relief, along with more significant improvements in intestinal microbiota levels and overall quality of life.
Hu et al. [20]	Male Sprague-Dawley (SD) rats selected to replicate the COPD model through cigarette smoke combined with LPS	Biostime Probiotic Powder by gavage; PSR-medium dose group was given 1.5 times the low dose, and the high-dose group was given three times the low dose	Experimental study	-	After high-dose PSR treatment, FEV0.3/FVC% and Cdyn significantly increased, while RI decreased. Compared to the COPD model, the Biostime Probiotic Powder group had a notably lower RI. Hematoxylin and eosin staining revealed reduced inflammatory cells, no thickening of the bronchial tube walls, and intact alveoli following treatment with PSR and Biostime Probiotic Powder. Compared to the model group, the levels of TNF-α, IFN-γ, IL-1β, IL-4, and IL-10 were normalized in both the PSR and Biostime Probiotic Powder groups. Additionally, mRNA and protein expressions of TLR4 and NF-κB were significantly reduced after treatment with PSR and Biostime Probiotic Powder.
Ya Li et al. [21]	Sprague Dawley (SD) rats exposed to tobacco smoke	Aminophylline (27 mg/kg/d); Probiotics (0.9×10^10^CFU/kg/d, Pure Encapsulations); BJF (12.42 g/kg/d) for 13 weeks	Experimental study	12	BJF improved pulmonary function and reduced lung inflammation. Additionally, BJF treatment altered the gut microbiota composition, significantly increasing the abundance of Firmicutes and the Firmicutes-to-Bacteroides ratio, and boosting SCFA levels, including acetate, butyrate, and propionate. Conversely, the abundance of Bacteroidetes, Proteobacteria, Spirochaetes, Clostridiaceae, and Treponema decreased following BJF administration. BJF also lowered the gene and protein expression of NLRP3, Caspase-1, IL-8, and IL-1β, while increasing GPR43 expression.
Karim et al. [22]	Male, 63-73 years old COPD patients	Vivomixx 112 billion*, one capsule a day for 16 weeks	A randomized, double-blind controlled trial	53 placebo, 51 probiotic group	Probiotics decreased plasma levels of zonulin, claudin-3, and CAF22, and improved HGS, gait speed, and SPPB scores. They also lowered plasma levels of C-reactive proteins and 8-isoprostane, which are markers of systemic inflammation and oxidative stress. Correlation analysis showed varying degrees of association between plasma biomarkers and sarcopenia indices. Although there was a statistical trend, a reduction in the prevalence of sarcopenia was not observed in the probiotic group.
Koning et al. [24]	Patients aged 18-80 with moderate-to-severe COPD who had an acute exacerbation of COPD and were treated with antibiotics	5 grams of a multispecies probiotic (Ecologic® AAD) twice daily for 14 days, starting simultaneously with the antibiotic treatment	Randomized double-blind controlled trial	13 placebo, 17 probiotic group	Compared to placebo, probiotic intake did not affect the analyzed biomarkers, except for a significant decrease in WBC at day 7. While no other differences were noted between the two groups, changes were observed within the probiotic group, including a decrease in sCD14 and increases in MCP‐1 and IL‐8. In the placebo group, a decrease in IL‐6 was observed.
He et al. [25]	Participants aged 20 years or older	Participants were classified into three groups according to the Sanders' dietary live microbe classification system: low, medium, and high dietary live microbe groups	Cross-sectional study	3513 (low-dietary live microbe), 3762 (medium dietary live microbe), 2516 (high dietary live microbe)	After adjusting for confounders, participants with a high intake of dietary live microbes had a lower prevalence of COPD compared to those with a low intake. No significant association with COPD was observed in the medium or low dietary live microbe groups. This inverse relationship was particularly noticeable among smokers, females, individuals aged 40-60 years, and those who were non-obese.
Lai et al. [26]	C57BL/6 COPD-induced mice (8–10 weeks old)	Oral treatment of *Pg* MTS01 (2×10^8^ colony-forming units per day per mouse)	Experimental study	3-17	The makeup of gut microbiota has a significant impact on the development of COPD caused by cigarette smoke, and fecal microbiota transplantation can reverse the progression of COPD. *Pg* was isolated and found to improve COPD by reducing inflammation in the intestines, boosting mitochondrial and ribosomal functions in the colon, correcting abnormal host amino acid metabolism in the blood, and mitigating lung inflammation. The lipopolysaccharides (LPS) from *Pg* have anti-inflammatory effects and greatly improve COPD by inhibiting the toll-like receptor 4 (TLR4) signaling pathway.
Liu et al. [27]	Female specific-pathogen-free (SPF) ICR mice (age 8 weeks)	*Pedioccus pentosaceus* SMM914 (1××10^9^ CFU/mouse every two days) and *Lactiplantibacillus plantarum* subsp. *plantarum* control (Lp) (1 × 10^9^ CFU per mouse) for 2 weeks by oral gavage	Experimental study	3-10	SMM914 significantly alters the gut microbiota by increasing the abundance of probiotics that produce short-chain fatty acids and engage in antioxidant metabolism. At the same time, SMM914 promotes the synthesis of L-tryptophanamide, 5-hydroxy-L-tryptophan, and 3-sulfino-L-alanine, which enhances the tryptophan-melatonin pathway and raises levels of 6-hydroxymelatonin and hypotaurine in the lungs. This modulation boosts the production of endogenous anti-inflammatory factors, reduces macrophage polarization towards the M1 phenotype, and ultimately alleviates oxidative stress in mice with COPD.
Younus et al. [28]	Patients ages 30-70 years with COPD	Probiotic ampule (Enterogermina sachets containing *Bacillus clausii*) 2 billion daily for 6 months	Randomized controlled trial	40	One month after treatment, there was a significant difference in the number of exacerbations (73% in the treatment group vs. 45% in the placebo group). However, from the second month to the sixth month, no significant difference in exacerbations was observed between the two groups. There were no significant differences in CRP levels or FEV1 between the treatment and placebo groups before and after treatment. After treatment, the mean CAT score differed significantly between the treatment and placebo groups, with the placebo group showing a higher score.

Antibiotic prophylaxis

Antibiotics have historically been utilized for various respiratory conditions owing to their anti-inflammatory properties [29]. Recent research, however, has shifted focus toward exploring the therapeutic potential of prophylactic antibiotics through their impact on the gut microbiome. In the study by Lai et al. [26], which has been previously discussed, the authors revealed that the beneficial effects of antibiotics in treating chronic obstructive pulmonary disease (COPD) are mediated by alterations in gut microbiota composition. This was demonstrated by transferring fecal microbiota from antibiotic-treated mice to recipient mice, which led to a significant reduction in COPD symptoms in the recipients [26]. Therefore, in the management of COPD, antibiotics not only offer anti-inflammatory benefits but also show promise in modulating gut microbiome diversity and composition.

Several antibiotics have been extensively researched in the literature for their ability to alleviate COPD symptoms and prevent exacerbations. In a systematic review by Herath et al. [30], studies of individuals over the age of 65 years with moderate-to-severe COPD showed that continuous and intermittent prophylactic antibiotics caused a significant reduction in the number of participants experiencing COPD exacerbations and frequency. Given the specific demographics of the groups studied, the results may not be generalizable to other groups [30]. The antibiotics studied were azithromycin, erythromycin, clarithromycin, doxycycline, roxithromycin, and moxifloxacin; however, all studies used macrolides. Consequently, the positive effects can only be attributed to macrolide use at least three times weekly [30]. In another meta-analysis by Lee et al. [31] compiling studies on macrolides, quinolones, tetracyclines, penicillins, and more, the proportion of patients with exacerbations and a number of exacerbations also decreased with antibiotic treatment of patients with COPD and chronic bronchitis. These findings provide noteworthy evidence for the positive effects of antibiotics in patients with chronic pulmonary diseases.

The efficacy of macrolides is particularly highlighted by several meta-analyses demonstrating that long-term use of these antibiotics reduces both the number and frequency of COPD exacerbations compared to placebo [32-35]. Specifically, azithromycin therapy has been shown to lower exacerbation rates with both daily and intermittent dosing regimens, although a minimum duration of six months is required to achieve significant efficacy [26,27]. While one study indicated a reduction in hospitalization risk, three other studies did not find a significant difference in hospitalization frequency [32-35]. Additionally, a trend toward adverse events associated with macrolide therapy was observed across all reviewed meta-analyses, with statistical significance reported in a study by Cui et al. [34]. Therefore, while prophylactic use of macrolides may offer benefits in preventing COPD exacerbations, further large-scale clinical trials are necessary to better assess the balance between these benefits and the risk of adverse effects. 

Other meta-analyses have concentrated on comparing the effects of different antibiotics. In a study by Janjua et al. [26], macrolides emerged as the most effective in reducing COPD exacerbations, followed by quinolones, while tetracyclines did not demonstrate significant efficacy compared to placebo. Quality of life, as measured by the St. George's Respiratory Questionnaire (SGRQ), did not show significant improvement with any of these antibiotics [26]. Wang et al. [36] further supported these findings, showing that macrolides, such as erythromycin and azithromycin, were the most effective, with the number needed to treat ranging from four to seven. However, Therapleton et al. [37] investigated whether there were significant differences in efficacy or safety among various prophylactic antibiotic regimens-such as macrolide plus tetracycline versus macrolide alone, quinolone versus macrolide, quinolone versus tetracycline, and macrolide versus tetracycline-but did not find significant results. This lack of significant findings may be attributed to small sample sizes and short study durations. Overall, macrolides appear to offer the most promise for alleviating COPD based on the current literature and could be considered for routine clinical practice pending further rigorous clinical trials.

While the antibiotics covered in the literature appear to hold potential for routine prophylactic use, long-term antibiotic use does not come without side effects. Some studies did not find significant reports of adverse effects compared to placebo treatments [31,36,38]. However, most studies did conclude that antibiotic therapy, particularly when used long-term, was associated with the occurrence of antibiotic-resistant organisms, with the exception of Therapleton et al.'s study [31,34,36]. In some studies of macrolide antibiotics, while statistical significance was not achieved, there was a trend toward increased adverse events, such as gastrointestinal and cardiovascular reactions with treatment [32,33]. Cui et al. [34]'s findings on this matter did reach statistical significance for gastrointestinal reactions, liver function impairment, and hearing impairment with antibiotic use, and Herath et al. [30] found significant gastrointestinal events with moxifloxacin. Hearing impairment with azithromycin use was also found by Herath et al. [30] and Cao et al. [35] and was at least partially reversible in many cases. Cao et al. [35] found two times higher adverse effects with macrolides than control. Given the notable occurrence of adverse effects with antibiotics, further discussion and research is needed to determine which patients can tolerate treatment and optimal treatment duration. The characteristics of studies selected for this section have been summarized in Table 2.

**Table 2:** Selected studies investigating the role of prophylactic antibiotics in the management of COPD COPD - chronic obstructive pulmonary disease

Reference	Subjects	Treatment	Study design	Number of participants	Main results
Herath et al. [30]	Adults (aged 40 or over) with COPD presenting with 1 or more exacerbations in the previous year	Administration of an oral prophylactic antibiotic continuously or intermittently	Systematic review	3923	The use of continuous and intermittent prophylactic antibiotics provides a clinically significant benefit in reducing exacerbations for COPD patients. All studies involving these antibiotics used macrolides, so this benefit is specific to macrolide antibiotics administered at least three times per week. The effects of pulsed antibiotics are still unclear and need more research.
Lee et al. [31]	Patients with COPD and/or acute bronchitis	Antibiotic therapy for at least 3 months	Meta-analysis	3900	Antibiotic treatment significantly reduced both the proportion of patients experiencing exacerbations and the number of exacerbations per patient in each study. However, the reduction in the number of exacerbations per patient per year was not statistically significant. Adverse events occurred with similar frequency in both the antibiotic and placebo groups. Nonetheless, long-term use of antibiotics was associated with an increased incidence of antibiotic-resistant organisms.
Donath et al. [32]	Patient ages 65–75 with severe to very severe COPD	Prophylactic macrolide therapy	Meta-analysis	1677	Patients taking macrolides experienced a 37% relative risk reduction in COPD exacerbations compared to those receiving a placebo. Additionally, the risk of hospitalization was reduced by 21%, and the likelihood of having at least one COPD exacerbation was reduced by 68% with macrolide treatment. Although there was a trend towards decreased mortality and increased adverse events among those taking macrolides, these differences were not statistically significant.
Ni et al. [33]	Adults (older than 18 years of age) with a diagnosis of stable COPD	Prophylactic use of macrolides administered orally in appropriate daily doses of at least one time a week for a period of at least 3 months	Meta-analysis	1666	Macrolides have the potential to reduce the frequency of exacerbations in COPD patients, as demonstrated by both unweighted and weighted analyses. Subgroup analysis indicated that 6-12 months of erythromycin or azithromycin therapy could be effective. Additionally, within studies using 6-12 months of azithromycin, both daily and intermittent dosing regimens significantly lowered exacerbation rates. There were no significant differences in overall hospitalizations or all-cause mortality between the treatment and control groups. However, a tendency for increased adverse events was observed in the treatment groups.
Cui et al. [34]	Adults with a diagnosis of stable COPD but not AECOPD	Prophylactic use of macrolides administered orally at least one time a week for a period of at least 3 months	Meta-analysis	2151	Long-term macrolide treatment decreased both the total number of patients experiencing one or more exacerbations and the rate of exacerbations per patient per year. Subgroup analyses indicated that a minimum duration of 6 months was necessary for both azithromycin and erythromycin to show efficacy. While macrolide therapy had some positive effect on the SGRQ total score, it did not reach clinical significance. The frequency of hospitalizations did not differ significantly between the treatment and control groups. However, chronic azithromycin use was associated with a higher likelihood of adverse events.
Cao et al. [35]	Patients with a diagnosis of stable COPD	Long-term use of macrolides	Meta-analysis	2939	Patients taking macrolides experienced a 23% relative risk reduction in COPD exacerbations compared to those on placebo. The median time to the first exacerbation was significantly extended for patients receiving macrolides. Subgroup analysis revealed that erythromycin was beneficial, while older patients were less responsive to macrolide treatment.
Wang et al. [36]	COPD patients aged >18 years and have a well-defined diagnosis of COPD and evidence of persistent airflow limitation	Prophylactic antibiotics given for a minimum period of 12 weeks	Meta-analysis	3683	Prophylactic antibiotics significantly decreased both the frequency of exacerbations and the number of patients experiencing one or more exacerbations. Erythromycin and azithromycin were found to be the most effective, with the number needed to treat ranging from four to seven. Additionally, prophylactic antibiotics notably improved quality of life. In six studies, the time to the first exacerbation was extended, though one study showed conflicting results. There were no significant changes in hospitalization rates or adverse events. Furthermore, no substantial differences were observed in lung function, bacterial load, or airway inflammation. However, there was a significant increase in antibiotic-resistant strains.
Therapleton et al. [37]	Patients of mean age 68 years, with moderate‐severity COPD who had between 2-5 exacerbations in previous 2 years	Antibiotic treatment duration of 12 to 13 weeks	Systematic review	391	It remains unclear if one antibiotic is more effective than another in reducing exacerbations or improving quality of life. In a 13-week study, no serious side effects or deaths were reported for moxifloxacin, azithromycin, or doxycycline. Another study, with a 12-week treatment period followed by 48 weeks of follow-up, found similar rates of serious side effects between the combined antibiotic and single antibiotic groups. However, in this study, five deaths occurred in the combined treatment group, compared to three in the single treatment group.
Janjua et al. [38]	Adults with COPD of mean age 64 to 73 years	Macrolide, tetracycline, or quinolone	Meta-analysis	3405	Compared to placebo, long-term use of macrolides (ranked highest) seemed to offer advantages in extending the time to the next exacerbation, enhancing quality of life, and reducing serious adverse events. No distinct benefits were noted with quinolones or tetracyclines. Additionally, antibiotic resistance emerged as a concern, though it could not be thoroughly evaluated in this review.

Dietary fiber

The breakdown of dietary fiber by gut microbes generates short-chain fatty acids (SCFAs) that may have protective effects against lung inflammation in patients with COPD [39]. Consequently, increasing fiber in the diet of COPD patients may reduce inflammation through restoring gut eubiosis. In a prospective cohort study by Szmidt et al. [40], long-term high dietary fiber intake, particularly from cereal and fruits, was shown to be associated with a 30% lower risk of COPD. People with any history of smoking who had low long-term fiber intake had a higher risk of COPD than never-smokers with high fiber intake [40]. However, the confounding variables of smoking and fiber intake question the validity of these findings. Further, as the study was performed only on women, the generalizability of these findings to the whole population needs further investigation. Accordingly, another prospective cohort study by Kaluza et al. [41], this time studying Swedish men, also found an inverse association between high dietary fiber intake and COPD incidence in current or ex-smokers but not in never-smokers. However, given the observational nature of these study designs, the possibility of misclassification of the level of fiber intake cannot be ruled out. People who consume more fiber may also have been consuming other beneficial nutrients that act as confounding variables in these studies [41]. Longitudinal randomized controlled trials may address some of these factors for more reliable results.

Varraso et al. [42] published another prospective study of American men and women and arrived at similar conclusions as the previous studies, but the findings were primarily only significant in women. The authors here also found that it was specifically high fiber associated with cereal that may be reducing the risk of developing COPD [42]. In a systematic review by Wald et al. [43], while the authors did not pool the data from their literature search due to methodological diversity, they found general associations between higher fiber intake and reduced risk of COPD, better lung function, and reduced respiratory symptoms. An investigation by Valisoltani et al. [44] also found an inverse correlation between COPD risk and intake of total fiber, cereal fiber, and fruit fiber, while the significance of vegetable fiber was not established. A 10-gram increase in total fiber, cereal fiber, or fruit fiber reduced the risk of COPD by 26%, 21%, and 37%, respectively. However, as the Risk of Bias in Non-randomized Studies - of Exposures (ROBINS-E) bias assessment tool reported a moderate risk of bias for the studies analyzed, further research is needed to validate these findings [44]. These studies direct future investigations to also investigate which sources of fiber would be the most effective and optimal for therapeutic use in COPD.

When considering implementing new treatments into clinical practice, extensive research is needed to determine which populations of people would benefit from the intervention. Jin et al. [45] looked further into dietary fiber intake in middle-aged and elderly populations. While Varraso et al. [42] found stronger associations between high dietary fiber intake and reduced incidence of COPD in women, Jin et al.'s [45] findings showed that the populations where this association was strongest in their study were men, middle-aged individuals, those with BMI less than 30 kg/m2, smokers, and alcohol consumers. Given this discrepancy in these two studies, further evaluation is needed to determine the populations of people in which increasing fiber intake would have the greatest benefit in reducing the risk of COPD. Randomized controlled trials and larger-scale cohort studies may help achieve this. Jin et al. also found that, specifically, a dietary fiber intake of at least 15.10 g/d was needed to effectively reduce the prevalence of COPD. Mediation analysis showed that white blood cell (WBC) count was involved in mediating the association between dietary fiber and COPD prevalence [45]. This study lays a good foundation for future research into the mechanism of action of fiber in COPD. 

Several studies have also sought to find associations between COPD and other dietary nutrients in addition to fiber. In a cross-sectional Korean study by Kim et al. [46], carbohydrates, protein, fiber, thiamin, riboflavin, niacin, and vitamin C intake were found to be associated with decreased COPD severity in elderly men over 60 years old. No statistical significance was found in women. In another retrospective Korean study of healthy subjects by Jung et al. [47], the authors hypothesized that the change in age-related dietary fiber intake affects lung function. They found new airflow limitations in 48 out of 1439 subjects at the five-year follow-up, primarily of whom were men and 22.9% of whom were current smokers. Through calculations of odds ratios, the authors also concluded that decreased intake of fiber, vitamin C, and folic acid was associated with newly developed airflow limitation [47]. Due to these findings being in specific Korean populations, it is worth further investigating whether COPD patients of different ethnicities have differing responses to the intake of fiber and other nutrients. In another systematic review by Seyedrezazadeh et al. [48], a higher intake of fruits, dietary fiber, and fish was found to reduce the risk of COPD, while no association was observed with vegetables, n-3 fatty acids, vitamin E, and β-carotene. Future studies may also investigate whether there is any correlation between increased intake of certain nutrients and risk of developing COPD.

Hain [49] explored the synergistic effects of dietary fiber in conjunction with omega-3 and omega-6 fatty acids on COPD. The study confirmed an association between dietary fiber and COPD status, cough, and chronic bronchitis. However, no significant links were found between omega-3 or omega-6 intake and COPD or respiratory outcomes. Notably, the study revealed that the impact of fiber on COPD outcomes was influenced by omega-6 intake and the omega-6:3 ratio. Specifically, lower omega-6 intake was associated with a higher likelihood of COPD, irrespective of fiber intake levels. Conversely, higher omega-6 intake was linked to a lower likelihood of COPD, regardless of fiber intake. Similar trends were observed with the omega-6:3 ratio [49]. These findings provide preliminary evidence for the interaction between dietary fiber, omega-6, and the omega-6:3 ratio in affecting COPD status. Further experimental research is needed to clarify the mechanisms underlying these interactions.

A randomized controlled trial by Miao et al. [50] is the only paper found during the literature search that actually directly evaluated the therapeutic potential of dietary fiber supplementation in patients with COPD. The study divided participants into a trial group that received 6 grams of sugarcane bagasse dietary fiber in addition to conventional COPD treatment and a control group that received a placebo in addition to conventional COPD treatment for 30 days. While clinical symptoms and severity of dyspnea were significantly improved with both fiber supplementation and placebo, no difference was seen between fiber and placebo treatment. However, when it comes to quality of life as determined by the SGRQ, there was a greater reduction in subscales of activity, effect, and total score in the fiber group than in the control group. The results suggest that sugarcane bagasse has the potential to improve the quality of life in patients with COPD when taken as adjunctive therapy along with conventional treatment. However, with no notable benefits seen in symptom reduction, further extensive clinical testing remains necessary for critically evaluating the benefits of incorporating fiber supplementation into the diets of patients with COPD [50]. Details of the studies selected for this section have been summarized inTable 3.

**Table 3:** Selected studies investigating the role of dietary fiber in the management of COPD COPD - chronic obstructive pulmonary disease

Reference	Subjects	Intervention or study variables	Study design	Number of subjects	Main results
Szmidt et al. [40]	Swedish women born during 1914-1948	Dietary fiber intake and COPD risk	Prospective cohort study	35,339	A long-term high intake of dietary fiber (≥ 26.5 vs. < 17.6 g/day) was linked to a 30% reduced risk of COPD. Specifically, fiber from cereals and fruits, but not vegetables, was associated with a lower risk of COPD. Current and former smokers with a low long-term total fiber intake (< 17.6 g/day) had a 33-fold and tenfold higher risk of COPD, respectively, compared to never-smokers with a high fiber intake (≥26.5 g/day).
Kaluza et al. [41]	Swedish men aged 45-79 years	Dietary fiber intake and COPD risk	Prospective cohort study	45,058	A strong inverse relationship between total fiber intake (≥36.8 vs. <23.7 g/day) and COPD was found in current and former smokers, but not in never-smokers. For cereal fiber, the hazard ratios (HR) comparing the highest to the lowest quintile were 0.62 for current smokers and 0.66 for ex-smokers. For fruit fiber, the HRs were 0.65 for current smokers and 0.77 for ex-smokers. For vegetable fiber, the HRs were 0.71 for current smokers and 0.92 for ex-smokers.
Varraso et al. [42]	American health professionals aged 30-75 years	Dietary fiber intake and COPD risk	Prospective cohort study	111,580	After adjusting for 11 factors (age, sex, smoking, energy intake, body mass index, US region, physician visits, physical activity, diabetes, and intake of omega-3 and cured meat), total dietary fiber intake was found to be negatively associated with the risk of newly diagnosed COPD. Among specific sources of fiber, only cereal fiber showed a significant independent association with newly diagnosed COPD, regardless of other fiber sources.
Wald et al. [43]	-	Dietary fiber intake, fatty acid intake, and COPD risk	Systematic review	-	Due to methodological differences, the data from the studies could not be combined. However, a higher intake of dietary fiber has consistently been linked to a lower risk of COPD, improved lung function, and fewer respiratory symptoms. In contrast, findings on the relationship between fatty acids and COPD are inconsistent.
Valisoltani et al. [44]	Adults aged 30-79 years	Dietary fiber intake and COPD risk	Meta-analysis	213,912	Analysis comparing the highest and lowest intakes revealed an inverse correlation with the intake of total fiber, cereal fiber, and fruit fiber, though this was not significant for vegetable fiber. Dose-response analysis indicated that a daily increase of 10 g in total dietary fiber, cereal fiber, or fruit fiber was associated with a 26%, 21%, and 37% reduction in COPD risk, respectively. The ROBINS-E tool assessed all cohort studies as having a moderate risk of bias. According to the NutriGrade tool, the credibility of findings related to total fiber, cereal fiber, and fruit fiber was low. There is insufficient scientific evidence to support the use of vegetable fiber in COPD management.
Jin et al. [45]	Participants aged >40 years	Dietary fiber intake and COPD risk	Prospective cohort study	7301	An increase in dietary fiber (DF) intake was significantly associated with a reduction in the prevalence of COPD, and DF intake was correlated with lung function indicators such as FEV1. Stratified analysis showed that higher DF intake significantly lowered the risk of COPD in males, individuals aged 40-59 years, those with a BMI ≤30 kg/m², smokers, and alcohol consumers. Restricted cubic spline analysis revealed that the critical threshold for DF intake's effect on COPD prevalence was 15.10 g/day; DF intake at or above this level effectively reduced COPD prevalence. Mediation analysis found that WBC count partially mediated the relationship between DF intake and COPD, accounting for 9.89% of the mediation effect.
Kim et al. [46]	Korean men and women aged >40 with COPD	Dietary carbohydrate, fat, protein, fiber, vitamin A, beta-carotene, retinol, thiamin, riboflavin, niacin, and vitamin C intake and COPD risk	Analytical cross-sectional study	702	In a model adjusted for covariates, vitamin A intake was positively associated with FEV1% in men. Intake of carbohydrates, proteins, fiber, thiamin, riboflavin, niacin, and vitamin C was significantly linked to reduced disease severity in elderly men (aged ≥60 years). In contrast, no significant associations were observed for these nutrients in women. Overall, the consumption of carbohydrates, proteins, fiber, thiamin, riboflavin, niacin, and vitamin C was associated with reduced severity of airway impairment in elderly men with COPD.
Jung et al. [47]	Adults with normal spirometry	Dietary fiber, folic acid, vitamin C, and COPD risk	Retrospective cohort study	1439	At the 5-year follow-up, new airflow limitations were identified in 48 subjects (3.3%), comprising 41 men (85.4%) and 11 current smokers (22.9%). After adjusting for age, sex, smoking history, and baseline FEV1/FVC, the odds ratios (OR) for new airflow limitations associated with a 10% decrease in daily recommended intake of fiber, vitamin C, and folic acid were 2.714, 1.083, and 1.495, respectively.
Seyedrezazadeh et al. [48]	Subjects over age 16	Dietary fish, fruits, vegetables, fiber, fatty acids, vitamin C, vitamin E, β-carotene, and COPD risk	Meta-analysis	-	The pooled relative risks (RRs) and confidence intervals for COPD, comparing the highest intake group to the lowest intake group, were 0.74 for fruit, 0.65 for dietary fiber, 0.71 for fish, and 0.89 for vitamin C. No significant association was found between COPD risk and the intake of vegetables, n-3 fatty acids, vitamin E, or β-carotene. However, an association was observed with n-6 fatty acids, with an RR of 1.06.
Hain [49]	US adults ≥40 years of age	Omega-3, omega-6, omega-6:3 ratio, fiber, COPD risk, lung function, respiratory morbidities	Analytical cross-sectional study	6938	Dietary fiber intake was found to be linked to COPD status, cough, and chronic bronchitis. However, there were no significant associations between omega-3 intake and COPD or respiratory outcomes, nor between omega-6 intake and COPD or respiratory outcomes. An interaction was observed between fiber quartiles and omega-6, as well as the omega-6:3 ratio, with COPD status.
Miao et al. [50]	Patients <80 years old with COPD	Sugarcane bagasse was made from fresh sugarcane into pills (a standard 1-g pill contained 0.5 g sugarcane bagasse/pill, plus the same other ingredients as in the placebo pill)	Randomized controlled trial	196	Post-treatment, both the trial group and the control group showed significant improvements in pulmonary clinical symptoms and the severity of dyspnea. However, there was no significant difference between the two groups regarding post-treatment pulmonary symptoms and mMRC scores. The trial group experienced a greater reduction in the SGRQ subscales for activity, impact, and total score compared to the control group. No significant differences were found in pre- and post-treatment safety variables in either group.

Fecal microbiota transplantation

Looking into the innovative therapies for COPD management, fecal microbiota transplantation (FMT) appears to be another promising area of research. In a mouse model study by Li et al. [51], fecal microbiota from healthy controls and COPD patients of varying GOLD stages were inoculated into recipient mice to induce changes in lung function. Fecal microbiota from the donors was also inoculated into mice in whom COPD was induced via smoke exposure. The authors found that the gut microbiome of COPD patients had a distinct composition and diversity compared to healthy individuals and was dominated by *Prevotella* species and lower levels of SCFAs. Recipient mice of COPD patients experienced higher lung inflammation, and when the mice were additionally also exposed to smoke, they had a decline in lung function, severe emphysematous changes, airway remodeling, and mucus hypersecretion [51]. While this is a solid foundational study showing how the gut microbiome is associated with COPD progression, further studies are needed to determine whether these changes observed are long-term or acute. Also, while this study establishes the relationship that FMT from unhealthy donors can induce COPD in healthy mice, the question remains: can FMT from healthy donors reverse COPD changes in diseased individuals?

To answer this question, Budden et al. [52] used a mouse model of cigarette smoke (CS)-induced COPD and FMT. The results were promising and showed that FMT alleviated COPD-associated inflammation, alveolar destruction, impaired lung function, gastrointestinal pathology, and systemic immune changes. These protective changes were additive to smoking cessation. Like the previous study, FMT of CS-associated microbiota in microbiome-depleted mice was sufficient to increase lung inflammation and suppress colonic immunity despite the absence of CS exposure. The authors used proteomics and metabolomics, which illustrated the downregulation of glucose and starch metabolism in CS-associated microbiota. As follows, supplementing mice and human patients with complex carbohydrates was able to improve COPD [52]. Jang et al. [53] also used a mouse model exposed to smoking and poly I:C for emphysema, and they found increased levels of Bacteroidaceae and Lachnospiraceae, SCFA metabolizers, in diseased mice with FMT from healthy donors and diseased mice supplemented with a high fiber diet. These were associated with a decrease in inflammation and inhibition of emphysema progression [53]. These studies highlight the intricate relationship between the gut microbiome and COPD pathogenesis, suggesting that modulation of the gut microbial composition through FMT holds therapeutic potential.

The research that has been published thus far is very preliminary and experimental. There remain several gaps in knowledge that must be addressed in future studies of FMT. Further elucidating the mechanisms of how FMT modulates the gut-lung axis and influences COPD pathogenesis is needed. FMT protocols must also be optimized for FMT administration, preparation methods, dosing regimens, and frequency of treatments depending on how long the effects of transplantation last. This could involve randomized controlled trials comparing different FMT protocols to identify the safest and most effective approach. Studies can also explore the concept of personalized FMT therapy based on the individual patient's microbiome composition. The long-term safety and adverse effects of FMT in humans also remain unknown. Addressing these research gaps can pave the way for the integration of FMT into clinical practice. The studies summarized in this section are outlined in Table 4.

**Table 4:** Selected studies investigating the role of FMT in the management of COPD FMT - fecal microbiota transplantation; COPD - chronic obstructive pulmonary disease

Reference	Subjects	Treatment	Study design	Sample size (N)	Main results
Li et al. [51]	Males aged 40-80 years with COPD diagnosed over 1 year ago; male C57BL/6 mice	fecal microbiota transplantation performed by a single oral administration of 100 μL per mouse every other day, for a total of 14 times in 28 days	Experimental study	15	The gut microbiome in COPD patients differed from that of healthy controls, showing distinct microbial diversity and composition, a Prevotella-dominated gut enterotype, and reduced levels of short-chain fatty acids. After 28 days of fecal transplantation from COPD patients, recipient mice showed increased lung inflammation. Furthermore, when mice were exposed to both fecal transplantation and biomass fuel smoke for a total of 20 weeks, they experienced more rapid declines in lung function, severe emphysematous changes, airway remodeling, and mucus hypersecretion.
Budden et al. [52]	Mouse model of cigarette smoke-induced COPD	Fecal microbial transfer	Experimental study; randomized-controlled trial	16 for randomized controlled trial; unknown for mouse model study	FMT mitigated key features of COPD, including inflammation, alveolar destruction, and impaired lung function, as well as gastrointestinal pathology and systemic immune alterations. Its protective effects were complementary to smoking cessation. Additionally, the transfer of cigarette smoke-associated microbiota following antibiotic-induced microbiome depletion was sufficient to increase lung inflammation and suppress colonic immunity even in the absence of cigarette smoke exposure. Disease features correlated with the relative abundance of members of the Muribaculaceae, Desulfovibrionaceae, and Lachnospiraceae families. Proteomics and metabolomics revealed a downregulation of glucose and starch metabolism in cigarette smoke-associated microbiota, and supplementation with complex carbohydrates improved disease outcomes in both mice and human patients.
Jang et al. [53]	Eight-week-old inbred female C57BL/6 mice	200 mg of fresh feces from control mice transplanted into recipient mice using oral gavage with 200 μL of resuspended feces twice a week at 3 and 4 weeks; SCFAs administered in drinking water at 76, 29, and 45 mM for 3 weeks	Experimental study	4-6	Smoking exposure increased the mean linear intercept. Both fecal microbiota transplantation (FMT) and a high-fiber diet (HFD) mitigated this increase. Mice undergoing FMT did not experience weight loss combined with smoking exposure. HFD reduced the number of macrophages and lymphocytes in bronchoalveolar lavage fluid. Levels of IL-6 and IFN-γ were lowered in both bronchoalveolar lavage fluid and serum. The TUNEL score was significantly reduced in mice receiving FMT or HFD, indicating decreased apoptosis. The families Bacteroidaceae and Lachnospiraceae increased with both FMT and HFD. FMT and HFD attenuated emphysema development through local and systemic inhibition of inflammation and alterations in gut microbiota composition, potentially offering a new approach for COPD treatment.

## Conclusions

There is strong evidence that gut dysbiosis influences COPD progression, and manipulation of the gut microbiome shows promise as a therapeutic alternative for managing this chronic illness. Prebiotics, probiotics, and synbiotics have shown potential in restoring gut dysbiosis and alleviating COPD progression. The administration of prophylactic antibiotics has demonstrated efficacy in reducing exacerbation frequency and severity, possibly by altering the composition of the respiratory microbiome via the gut-lung axis. Additionally, dietary fiber supplementation exhibits beneficial effects on gut microbial diversity and function, suggesting therapeutic benefits in COPD management. Furthermore, the latest insights into FMT are promising, although further research is needed to understand its efficacy and safety profile in COPD patients. More longitudinal studies with larger cohorts are needed to further investigate these findings and establish optimal treatment protocols in clinical practice.
